# SLC35A3 Remodels Site-Specific N-Glycosylation and Defines a Druggable Glycosylation Target in Hepatocellular Carcinoma

**DOI:** 10.1016/j.mcpro.2026.101672

**Published:** 2026-10-05

**Authors:** Qin Ouyang, Ke Gao, Xinwen Zhou, Jieyi Shi, Long Liao, Cun Wang, Shu Zhang

**Affiliations:** 1 Liver Cancer Institute, Zhongshan Hospital, Fudan University, Key Laboratory of Carcinogenesis and Cancer Invasion of Ministry of Education, Shanghai, China; 2 Institutes of Biomedical Sciences, Fudan University, Shanghai, China; 3 State Key Laboratory of Systems Medicine for Cancer, Shanghai Cancer Institute & Department of Liver Surgery, Renji Hospital, Shanghai Jiao Tong University School of Medicine, Shanghai, China

**Keywords:** SLC35A3, hepatocellular carcinoma, N-glycosylation, UDP-GlcNAc, glycoproteomics

## Abstract

Aberrant N-glycosylation contributes to hepatocellular carcinoma (HCC) progression, yet the upstream regulators that coordinate nucleotide-sugar transport and site-specific N-glycosylation remain poorly characterized. Here, we identified SLC35A3, a Golgi-resident UDP-GlcNAc transporter, as a glycosylation-related tumor suppressor in HCC. SLC35A3 was downregulated in HCC tissues, and its restoration suppressed cell proliferation and tumorigenicity. Mechanistically, SLC35A3 overexpression increased Golgi-resident UDP-GlcNAc levels, indicating enhanced substrate availability for glycosyltransferases. Intact N-glycopeptide profiling demonstrated that SLC35A3 overexpression reprogrammed site-specific N-glycan processing, characterized by a predominant reduction in high-mannose (HM) glycoforms and selective remodeling of fucosylated-sialylated (FS) glycoforms. Molecular docking identified poliumoside as a putative SLC35A3 binder with anti-tumor effect in HCC models. Together, our findings establish SLC35A3 as a critical node in Golgi-dependent N-glycan remodeling and highlight its potential as a target for pharmacological activation.

## Highlights

• SLC35A3 is downregulated in HCC and associates with poor prognosis.
• SLC35A3 restoration suppresses HCC growth in cells and xenografts.
• SLC35A3 restoration elevates Golgi UDP-GlcNAc and remodels N-glycosylation.
• Poliumoside is a candidate SLC35A3 binder with antitumor activity in HCC.

## Graphical Abstract

**Figure:**
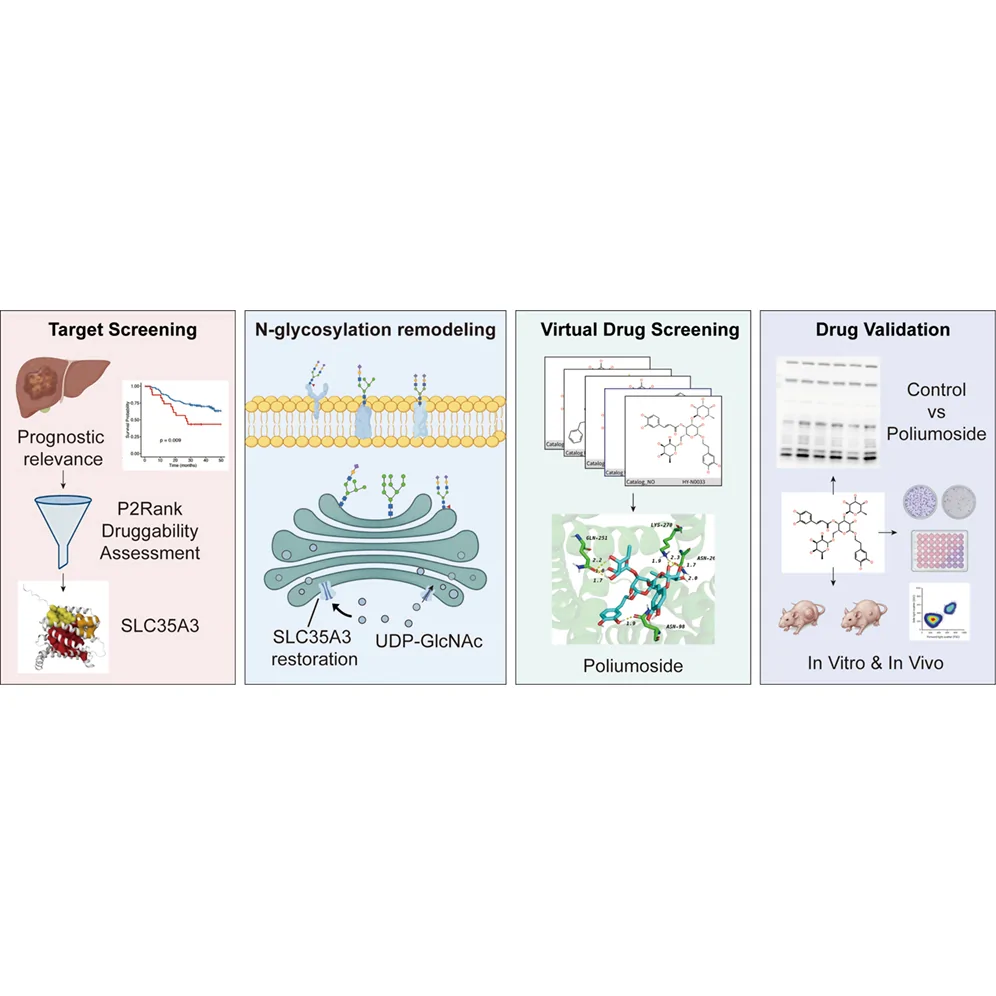

## In Brief

Our study identifies SLC35A3 as a tumor suppressor in HCC and shows that its restoration is associated with increased Golgi UDP-GlcNAc levels and selective remodeling of site-specific N-glycosylation. Molecular docking and functional screening nominate poliumoside as a candidate modulator of SLC35A3-associated glycosylation, providing a rationale for future pharmacological investigation.

Hepatocellular carcinoma (HCC) accounts for approximately 90% of primary liver cancers (1). Despite remarkable advances in HCC treatment, mortality rates continue to rise globally (2). Most patients are diagnosed at intermediate or advanced stages, when curative treatments such as surgical resection, ablation, or liver transplantation are no longer feasible (3). Although systemic therapies have expanded the treatment landscape of advanced HCC, therapeutic resistance and disease recurrence remain common (4). Therefore, elucidating the molecular mechanisms underlying HCC progression and translating these insights into therapeutic strategies remains a pressing priority.

Aberrant protein glycosylation is a hallmark of cancer, with altered glycan structures contributing to tumor progression, immune evasion and metastasis 5, 6. Protein N-glycosylation is arguably the most complex post-translational modification, owing to the non-template-driven nature of glycan biosynthesis that generates an enormous repertoire of glycoforms 7, 8. N-linked glycosylation is initiated in the endoplasmic reticulum, where a preassembled oligosaccharide precursor is transferred to asparagine residues within the consensus N-X-S/T sequon (X ≠ P) of nascent polypeptides by the oligosaccharyltransferase (OST) complex (9). Following initial glucose trimming and quality control in the endoplasmic reticulum, N-glycoproteins are transported to the Golgi apparatus, where their N-glycans undergo divergent maturation into retained high-mannose structures or further processed hybrid and complex-type glycans. This maturation is mediated by coordinated actions of glycosidases and glycosyltransferases and critically depends on the luminal availability of nucleotide-sugar donors (10).

Consequently, perturbations at any of these regulatory nodes—glycosidase trimming, glycosyltransferase branching, or nucleotide-sugar transport—can reshape glycosylation in a site-specific manner, generating substantial heterogeneity. Recent advances in mass spectrometry-based glycoproteomics have substantially improved the depth, accuracy, and throughput of site-specific glycan profiling, enabling large-scale characterization of intact glycopeptides in complex biological specimens 11, 12, 13, 14. However, elucidating how site-specific glycan microheterogeneity influences protein function and contributes to cancer progression remains a major challenge.

The clinical translation of aberrant glycosylation in cancer has been most prominent in biomarker discovery (15). These biomarkers have proven valuable for tumor detection, surveillance, and risk stratification but do not illuminate actionable targets (16). Rather than a passive byproduct of malignant transformation, aberrant glycosylation actively drives tumorigenesis, immune evasion, and metastasis. We reasoned that upstream regulators dysregulated in cancer are attractive, potentially druggable nodes whose restoration or inhibition could normalize pathogenic glycosylation and provide a mechanistic basis for therapeutic intervention.

## Experimental Procedures

### Experimental Design and Statistical Rationale

This study employed an integrative screening strategy to identify prognostically relevant and potentially druggable glycosylation-related targets in HCC. Public proteomic datasets were analyzed to prioritize dysregulated proteins associated with patient prognosis, followed by machine learning-based prediction of ligand-binding pockets and intersection with glycosylation-related genes. We next validated the tumor-suppressive role of the prioritized candidate, SLC35A3, in HCC cell lines and xenograft models. LC-MS/MS-based site-specific N-glycoproteomic profiling was performed to characterize N-glycosylation remodeling associated with SLC35A3 overexpression. Finally, structure-based virtual screening followed by functional validation was used to prioritize candidate small molecules that may modulate SLC35A3-associated glycosylation phenotypes, and the antitumor activity of the selected compound was evaluated in HCC models.

For the LC-MS/MS-based site-specific N-glycoproteomic analysis, three independent biological replicates were analyzed for each experimental group (n = 3 per group). Each biological replicate was derived from an independently prepared sample and was treated as the independent experimental unit for statistical analysis. Intact glycopeptides were identified using pGlyco 3.1 against the human UniProt database sp_human_202403, which contained 20,433 protein entries. All peptide, glycopeptide, and protein identifications were filtered using a 1% false discovery rate (FDR) cutoff.

All statistical analyses were performed using Prism 10 (GraphPad Software) and R software (v. 4.4.1). Quantitative data are reported as mean ± SD where indicated. Statistical tests were two-sided unless otherwise specified. Statistical methods for each analysis are detailed in the corresponding figure legends, with significant differences indicated by asterisks at the following thresholds: ∗*p* < 0.05, ∗∗*p* < 0.01, ∗∗∗*p* < 0.001, and ∗∗∗∗*p* < 0.0001.

### Tissue Microarray (TMA) Construction

Fresh HCC tissues and paired adjacent normal liver tissues (>2 cm from tumor margin) were collected from 50 patients who underwent surgical resection at Zhongshan Hospital, Fudan University. The inclusion criteria were (i) pathologically confirmed HCC; (ii) no preoperative chemotherapy or radiotherapy; and (iii) maximum tumor diameter >3 cm. The study received approval from the Research Ethics Committee of Zhongshan Hospital (B2021-350R). All human studies reported in this study were conducted in accordance with the Declaration of Helsinki. All tissues were fixed in 4% neutral buffered formalin for at least 24 h and sent to Servicebio Technology Co., Ltd (Wuhan, China) for TMA construction. Tissue cores of 2.0 mm in diameter were punched from representative areas and arrayed in a paired design. The TMA block was sectioned at 4 μm for subsequent immunohistochemical analysis.

### Immunohistochemistry

Immunohistochemistry was performed on formalin-fixed paraffin-embedded (FFPE) TMA sections (4 μm). After deparaffinization in CitriSolv (Fisher Scientific; a xylene substitute) and rehydration through graded ethanol (100%, 95%, 80%, 70%; 5 min each) to distilled water, antigen retrieval was performed by microwave heating in Tris-EDTA buffer (pH 9.0) for 15 min, followed by cooling to room temperature for 35 min. Slides were incubated with 3% H_2_O_2_ solution for 10 min to quench endogenous peroxidase activity at room temperature. Non-specific binding was blocked with 4% bovine serum albumin (BSA) and 2% normal donkey serum in TBS-T (0.05% Tween-20) for 1 h at room temperature. Sections were incubated with primary antibody [anti-SLC35A3, 1:100, rabbit polyclonal, Sino Biological, 203636-T08] overnight at 4 °C in a humidified chamber. After washing in TBS-T (3 × 5 min), sections were incubated with HRP-conjugated secondary antibody [donkey anti-rabbit IgG-HRP, 1:300, Jackson ImmunoResearch, 711-035-152] for 1 h at room temperature. Staining was visualized using 3,3′-diaminobenzidine (DAB) chromogen (Dako, K3468) for 1 to 2 min, counterstained with hematoxylin for 30 s, differentiated in hydrochloric acid-ethanol solution (Beyotime, C0161S) for 2 to 5 s, rinsed in running tap water for 5 min to blue the nuclei, dehydrated through graded ethanol (75%, 95%, 100%; 3 min each) and CitriSolv, and mounted with neutral balsam.

### Generation of Stable Cell Lines by Lentiviral Transduction

For stable SLC35A3 overexpression, the coding sequence of human SLC35A3 (NM_001271685.2) was cloned into the pLV[Exp]-Puro-EF1A lentiviral expression vector (VectorBuilder). Recombinant lentiviruses were generated by co-transfecting HEK-293T packaging cells with the pLV-EF1A-SLC35A3-Puro plasmid, psPAX2 packaging plasmid, and pMD2.G envelope plasmid using Lipofectamine 2000 (Cat. No. 11668019, Invitrogen). Viral supernatants were collected at 48 h, filtered through a 0.45 μm membrane, and concentrated. JHH7 and Huh7 cells were infected with lentivirus in the presence of 8 μg/ml polybrene. After 48 h, cells were selected with 2 μg/ml puromycin for 14 days. Stable overexpression was confirmed by western blotting.

### Western Blotting

Cell lysates were prepared using RIPA lysis buffer (Beyotime Biotechnology) supplemented with protease and phosphatase inhibitors. Protein concentrations were determined by the BCA assay (Beyotime Biotechnology). Equal amounts of protein (20 μg per lane) were separated by 10% SDS-PAGE and transferred onto PVDF membranes (Millipore). After blocking with Western Blocking Buffer (Cat. No. P0023B, Beyotime) for 30 min at room temperature, membranes were incubated overnight at 4 °C with primary antibodies against SLC35A3 (1:1000, PA5-42454, Invitrogen). After washing with TBST, membranes were incubated with HRP-conjugated secondary antibodies for 1 h at room temperature. Protein bands were visualized using an ECL chemiluminescence kit (Beyotime Biotechnology) and captured with a ChemiDoc imaging system (Bio-Rad). Densitometric quantification was performed using ImageJ software (NIH).

### Lectin Blotting

Cell lysates were prepared and separated by SDS-PAGE as described for Western blotting. After transfer to PVDF membranes, membranes were blocked with Carbo-Free Blocking Solution (Cat. No. SP-5040-125, Vector Laboratories) for 1 h at room temperature. Membranes were then incubated with biotinylated lectins (ConA, MAL-II, SNA, or AAL; Vector Laboratories) at room temperature for 30 min. After washing three times with TBST (5 min each), membranes were incubated with horseradish peroxidase-conjugated avidin D (Vector Laboratories) at room temperature for 30 min. Following three additional TBST washes, signals were developed using a standard ECL chemiluminescence kit. Blots were captured using a ChemiDoc imaging system (Bio-Rad).

### Cell Lines and Cell Culture

Human HCC cell lines (Huh7, JHH7) and HEK293T cells were procured from the Type Culture Collection of the Chinese Academy of Sciences in Shanghai, China. Authentication of these cells was conducted via Short Tandem Repeat (STR) analysis, and *mycoplasma* contamination was confirmed as negative using the *Mycoplasma* Detection Kit (MP0050, Sigma-Aldrich). All cells were cultured in DMEM supplemented with 10% fetal bovine serum plus 1% streptomycin and penicillin at 37 °C in a humidified atmosphere with 5% CO_2_.

### Cell Counting Kit-8 (CCK-8) Assay and Colony Formation

Cell viability was assessed using the CCK-8 assay (Dojindo Molecular Technologies) according to the manufacturer's protocol. Cells were seeded into 96-well plates at a density of 2 × 10^3^ cells per well in 200 μl of complete medium and cultured at 37 °C in the presence of 5% CO_2_ for 4 h, 24 h, 48 h. At each time point, CCK-8 reagent (10%, v/v) was added and incubated for 2 h, and absorbance at 450 nm was measured using a Synergy Neo2 multimode microplate reader (BioTek).

For the colony formation assay, 2 × 10^3^ cells were seeded into 6-well plates and cultured for 2 weeks. Colonies were fixed with 4% paraformaldehyde for 10 min, stained with 0.1% crystal violet (Solarbio) for 20 min.

### Xenograft Tumorigenesis Assay

The 5-week-old male BALB/c nude mice were purchased from the GemPharmatech Co., Ltd (Jiangsu, China) and maintained in specific pathogen-free (SPF) conditions. All animals were acclimated for 1 week before experiments. The 5 × 10^6^ JHH7 cells (wild-type or overexpressed with SLC35A3) in 100 μl PBS were subcutaneously inoculated at the flanks of randomly grouped (10 mice per group) nude mice. Tumor sizes were measured every 2 days with an electronic calliper, and tumor volumes were calculated. All mice were euthanized, and tumors were harvested and photographed 2 to 3 weeks after inoculation.

To study the therapeutic effects of poliumoside in preclinical tumor models, 5 × 10^6^ JHH7 cells were suspended in 100 μl PBS and injected subcutaneously into nude mice. When the tumor size reached approximately 100 mm^3^, the mice were randomly divided into two groups and 20 mg/kg of poliumoside or equivalent volume of vehicle was injected intraperitoneally on days 14, 16, 18 and 20 after tumor cell inoculation (10 mice per group). Tumor volumes and body weights were monitored throughout the study. At the endpoint, mice were euthanized, and tumors were harvested, weighed and photographed. All animal experiments were reviewed and approved by the Animal Ethics Committee of Zhongshan Hospital, Fudan University (protocol number: 2026-010).

### Flow Cytometric Analysis of Apoptosis

JHH7 cells seeded at 5 × 10^5^ per well were treated with poliumoside at 10 μM for 48 h. Cells were then harvested, washed twice with ice-cold PBS, and resuspended in Annexin V Binding Buffer (Cat. No. 422201, BioLegend). After staining with fluorochrome-conjugated Annexin V at room temperature in the dark for 20 min, cells were washed once with PBS. Propidium iodide (PI) was then added at a 1:100 dilution immediately prior to flow cytometric analysis. Early apoptotic (Annexin V+/PI−) and late apoptotic/necrotic (Annexin V+/PI+) populations were analyzed using a CytoFLEX S flow cytometer (Beckman Coulter). Data were acquired within 10 min of PI addition to avoid fluorescence quenching.

### Quantification of UDP-GlcNAc in Golgi Apparatus

Isolation of the Golgi apparatus from JHH7 cells using the Minute Golgi Apparatus Enrichment Kit (Cat. No. GO-037, Invent Biotechnologies) following the manufacturer's protocol. Briefly, 3 × 10^7^ cells were collected, washed twice with ice-cold PBS, and resuspended in 550 μl Buffer A supplemented with protease inhibitor cocktail. The cell suspension was vigorously vortexed for 30 s, transferred to a pre-chilled filter cartridge, and centrifuged at 16,000×*g* for 30 s. The filtrate was further centrifuged at 16,000×*g* for 30 min at 4 °C, and the resulting supernatant (400 μl) was mixed with an equal volume of Buffer B, incubated on ice for 15 min, and centrifuged at 8000×*g* for 5 min. The pellet was resuspended in 200 μl Buffer A, mixed with 100 μl Buffer C, and incubated on ice for 20 min to precipitate the Golgi fraction. After centrifugation at 8000×*g* for 10 min, the pellet containing enriched Golgi was resuspended in 100ul Minute Non-Denatured Protein Solubilization Reagent (Cat. No. WA-010, Invent Biotechnologies) for downstream ELISA analysis.

For UDP-GlcNAc quantification, Golgi-enriched fractions were analyzed using a Human UDP-GlcNAc ELISA Kit (Cat. No. NLH3827, NEWLF). Briefly, standards and samples were prepared according to the manufacturer's instructions. For sample measurement, 40 μl of sample diluent and 10 μl of Golgi fraction (final dilution, 5-fold) were added to each well, followed by 100 μl of enzyme conjugate per well. The plate was sealed and incubated at 37 °C for 60 min. After washing five times with diluted wash buffer, 50 μl of chromogen solution A and 50 μl of chromogen solution B were added to each well, and the plate was incubated at 37 °C in the dark for 15 min. The reaction was terminated by adding 50 μl of stop solution to each well. Absorbance at 450 nm was measured immediately using a microplate reader, and UDP-GlcNAc concentrations were calculated from a standard curve. Values were normalized to the total protein content of each Golgi fraction and are presented as ng/mg protein.

### Sample Preparation and Enrichment of Intact N-Glycopeptides

Cell pellets were lysed in Western and IP cell lysis buffer (Cat. No. P0013, Beyotime Biotechnology) supplemented with 1% (v/v) protease inhibitor cocktail (Roche) on ice for 30 min. The lysates were centrifuged at 14,000×*g* for 10 min at 4 °C, and the supernatants were collected. Protein concentration was determined using the BCA Protein Assay Kit (Cat. No. P0009, Beyotime Biotechnology). For each sample, 1 mg of protein was reduced with 10 mM DL-dithiothreitol (DTT) at 37 °C for 60 min and alkylated with 20 mM iodoacetamide (IAA) at room temperature in the dark for 30 min. The reduced and alkylated proteins were precipitated by adding six volumes of ice-cold acetone and incubating at −20 °C for at least 3 h. After centrifugation at 14,000×*g*, the protein pellets were dissolved in 8 M urea to a final concentration of 2 μg/μl with vortexing at 4 °C. The samples were then diluted 10-fold with 50 mM ammonium bicarbonate (ABC) and digested with trypsin at 37 °C overnight. The digestion was terminated by heating in a boiling water bath for 10 min.

Intact N-glycopeptides were enriched by zwitterionic hydrophilic interaction liquid chromatography (ZIC-HILIC). Briefly, ZIC-HILIC micro-columns were prepared by packing ZIC-HILIC particles (Merck Millipore) onto C8 disks (3M Empore) fitted at the bottom of 200 μl pipette tips. The columns were pre-equilibrated with 200 μl of loading buffer (80% acetonitrile, 1% trifluoroacetic acid) and centrifuged at 2000×*g* for 3 min (repeated twice). For each sample, 1 mg of tryptic peptides was dissolved in loading buffer to a final concentration of 5 μg/μl and loaded onto the ZIC-HILIC column. The column was centrifuged at 2000×*g* for 5 min, and the flow-through was reloaded onto the same column. This loading step was repeated four times to ensure complete binding of glycopeptides. Non-glycopeptides were removed by washing with 800 μl of wash buffer (80% acetonitrile, 1% trifluoroacetic acid). Bound glycopeptides were sequentially eluted with 200 μl of elution buffer 1 (0.1% trifluoroacetic acid), 200 μl of elution buffer 2 (50 mM ammonium bicarbonate), and 200 μl of elution buffer 3 (50% acetonitrile). The combined eluates were vacuum-dried and stored at −20 °C prior to LC-MS/MS analysis.

### LC-MS/MS Analysis of Intact N-Glycopeptides

Dried glycopeptides were reconstituted in solvent A (2% acetonitrile, 0.1% formic acid) and analyzed on a Thermo Orbitrap Exploris 480 mass spectrometer coupled with an EASY-nLC 1200 system (Thermo Fisher Scientific). Peptides were separated on a reversed-phase C18 analytical column (150 mm × 75 μm, 3 μm, Thermo Scientific) at a flow rate of 300 nl/min. Mobile phase A consisted of 0.2% formic acid in water, and mobile phase B consisted of 0.2% formic acid in acetonitrile. The gradient program was as follows: 5 to 40% solvent B over 90 min, followed by an increase to 90% B in 5 min, held for 5 min, and re-equilibration at 5% B for 5 min. Total ion chromatograms (TICs) were recorded to monitor the chromatographic separation (Supplementary Fig. S1).

For full-scan MS, spectra were acquired over m/z 350-1600 at a resolution of 120,000 with an automatic gain control (AGC) target of 3 × 10^5^. For tandem MS (MS/MS), precursor ions were isolated in the quadrupole with a 1.6 Th isolation window, fragmented by higher-energy collisional dissociation (HCD) at a normalized collision energy (NCE) of 30%, and detected in the ion trap using rapid scan mode. The MS/MS AGC target was set to 1 × 10^4^ with a maximum injection time of 60 ms. Only precursor ions with charge states 2 to 5 were selected for fragmentation. Dynamic exclusion was set to 30 s, and monoisotopic precursor selection was enabled with a precursor mass tolerance of 10 ppm.

### Database Search and Quantification

Intact N-glycopeptide identification was performed using pGlyco 3.1 against the human UniProt database. The search parameters were as follows: precursor and fragment ion mass tolerances of ±5 ppm and ±20 ppm, respectively; trypsin as the proteolytic enzyme with cleavage at lysine (K) and arginine (R); maximum of two missed cleavages; fixed modification of carbamidomethylation (C); and variable modifications of oxidation (M) and acetylation (protein N-terminal). Glycan database search was performed using the built-in pGlyco N-glycan database. Global protein quantification was performed using PEAKS software, and intact N-glycopeptide quantification was performed using pGlycoQuant based on the pGlyco 3.1 identification results.

### Molecular Docking

The AlphaFold-predicted human SLC35A3 structure (AF-Q9Y2D2-F1) was downloaded and prepared using the Protein Preparation Wizard in Schrödinger Maestro v12.8. Hydrogen atoms were added, bond orders were assigned, and energy minimization was performed using the OPLS2005 force field. Potential ligand-binding pockets were predicted using the SiteMap module, and Site 1, which showed the highest SiteScore (1.088), was selected for receptor grid generation. The grid box was centered on Site 1 with a size of 20 Å × 20 Å × 20 Å.

The MCE Bioactive Compound Library (25,300 compounds) and MCE 80K Diversity Library (80,000 compounds) were prepared using the LigPrep module. Virtual screening was performed using the Glide module in a hierarchical manner, comprising high-throughput virtual screening (HTVS), standard precision (SP), and extra precision (XP) docking. For the Bioactive library, the top 15% of compounds from HTVS were advanced to SP docking, and the top 15% from SP were subsequently subjected to XP docking. For the Diversity library, the top 10% of compounds were retained after each screening stage. The top 200 compounds from each library were exported for further analysis.

## Results

### Proteomic Screening and Druggability Prediction Identify SLC35A3 as a Glycosylation-Related Candidate Target in HCC

To identify prognostically relevant candidate targets in HCC, we analyzed two independent hepatitis B virus-related HCC proteomic cohorts, including the CPTAC-HCC cohort (17) and the Zhongshan-HCC cohort (18). Given that therapeutic restoration or reactivation of tumor-suppressive proteins has been proposed as an effective strategy to recover antitumor functions (19), we focused on proteins that were significantly downregulated in HCC tissues compared with adjacent normal liver tissues and whose low expression in tumor tissues was associated with poor patient prognosis. Application of these criteria identified 2055 candidate proteins in the CPTAC-HCC cohort and 473 candidate proteins in the Zhongshan-HCC cohort, and intersection of these two sets yielded 305 overlapping prognosis-associated proteins, as summarized in Supplementary Table S1. To further enrich for proteins biologically relevant to HCC, HCC-related genes were retrieved from the GeneCards database (keyword: "hepatocellular carcinoma"), and the top 80% ranked by relevance score (n = 8311) were retained and intersected with the 305 overlapping prognosis-associated proteins, yielding 162 candidate targets (Supplementary Table S1), as depicted in the overall study workflow (Fig. 1).

**Fig. 1:**
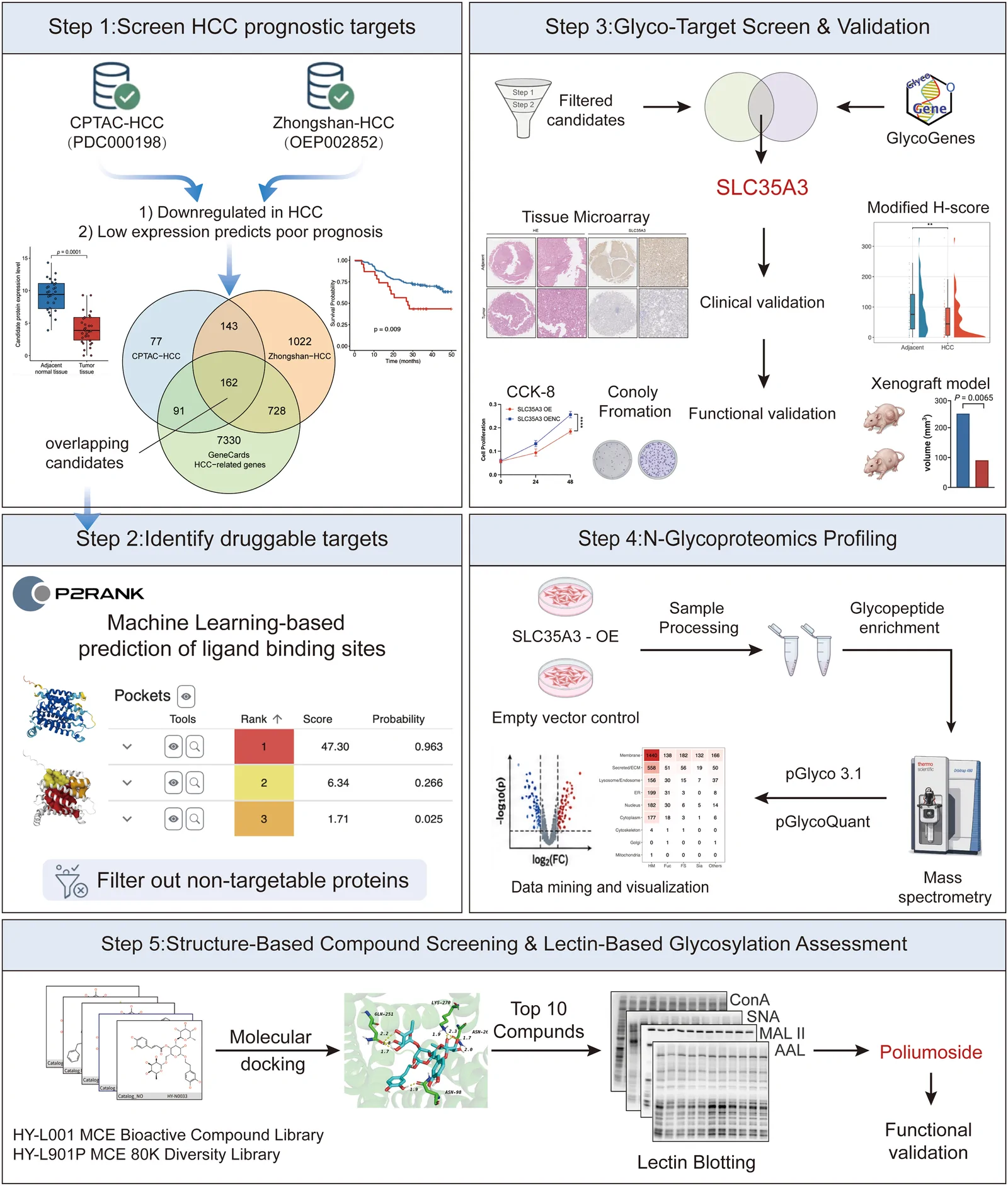
**Integrative workflow for the identification of SLC35A3 as a glycosylation-related therapeutic target in HCC**.

Then, we evaluated the structural ligandability of the candidate proteins using P2RANK, a machine learning–based method for predicting ligand-binding pockets (20). AlphaFold-predicted structures for the 162 candidate targets were retrieved using AlphaFold Extractor for subsequent P2Rank-based ligand-binding pocket prediction. In total, P2Rank identified 1095 putative ligand-binding pockets across 150 proteins (Supplementary Table S2). To prioritize candidates with high-confidence predicted ligand-binding sites, we retained proteins harboring at least one P2Rank-predicted pocket with a calibrated pocket probability greater than 0.9. Based on this criterion, 49 proteins were classified as high-confidence ligandable candidates and retained for downstream prioritization.

Aberrant glycosylation has been widely implicated in cancer progression, contributing to tumor cell signaling, invasion, metastasis, and immune interactions (21). Therefore, to identify ligandable targets with potential relevance to glycosylation biology, we intersected the structurally ligandable protein candidates with 222 glycosylation-related genes curated from the GlycoGene Database. Together, this integrative analysis identified SLC35A3 as a potential prognostic, druggable, glycosylation-related target in HCC.

### SLC35A3 is Downregulated in HCC, and Its Overexpression Suppresses HCC Cell Proliferation and Tumorigenicity

SLC35A3 was significantly downregulated in HCC tissues compared with adjacent normal liver tissues in both the CPTAC-HCC and Zhongshan-HCC cohorts, and its low expression was associated with poor patient prognosis (Fig. 2, *A* and *B*; Supplementary Fig. S2, *A* and *B*). This expression pattern was further validated in independent clinical HCC specimens. Western blot analysis of six paired clinical specimens revealed significantly lower SLC35A3 levels in HCC tissues than in matched adjacent normal liver tissues (Fig. 2*C*). We also constructed a paired TMA comprising HCC tissues and matched normal liver tissues from 50 patients. H&E staining confirmed the histological distinction between tumor and paired normal liver tissue cores. Representative SLC35A3 immunohistochemical staining showed weaker DAB signal in HCC tissues than in paired normal liver tissues (Fig. 2*D*). To quantitatively evaluate SLC35A3 staining in all 50 paired samples, we used QuPath to perform automated digital image analysis of TMA cores (22). Both the H-score and the Allred score were calculated for each tissue core based on DAB-positive cell percentages and staining intensities (Supplementary Table S3). Consistent with the representative images, QuPath-derived modified H-scores were significantly lower in HCC tissues than in adjacent normal liver tissues (Fig. 2*E*). The result showed that HCC tissues exhibited reduced positive staining across all intensity categories, together with a lower Allred score, compared with adjacent normal liver tissues (Supplementary Fig. S2, *C* and *D*).

**Fig. 2:**
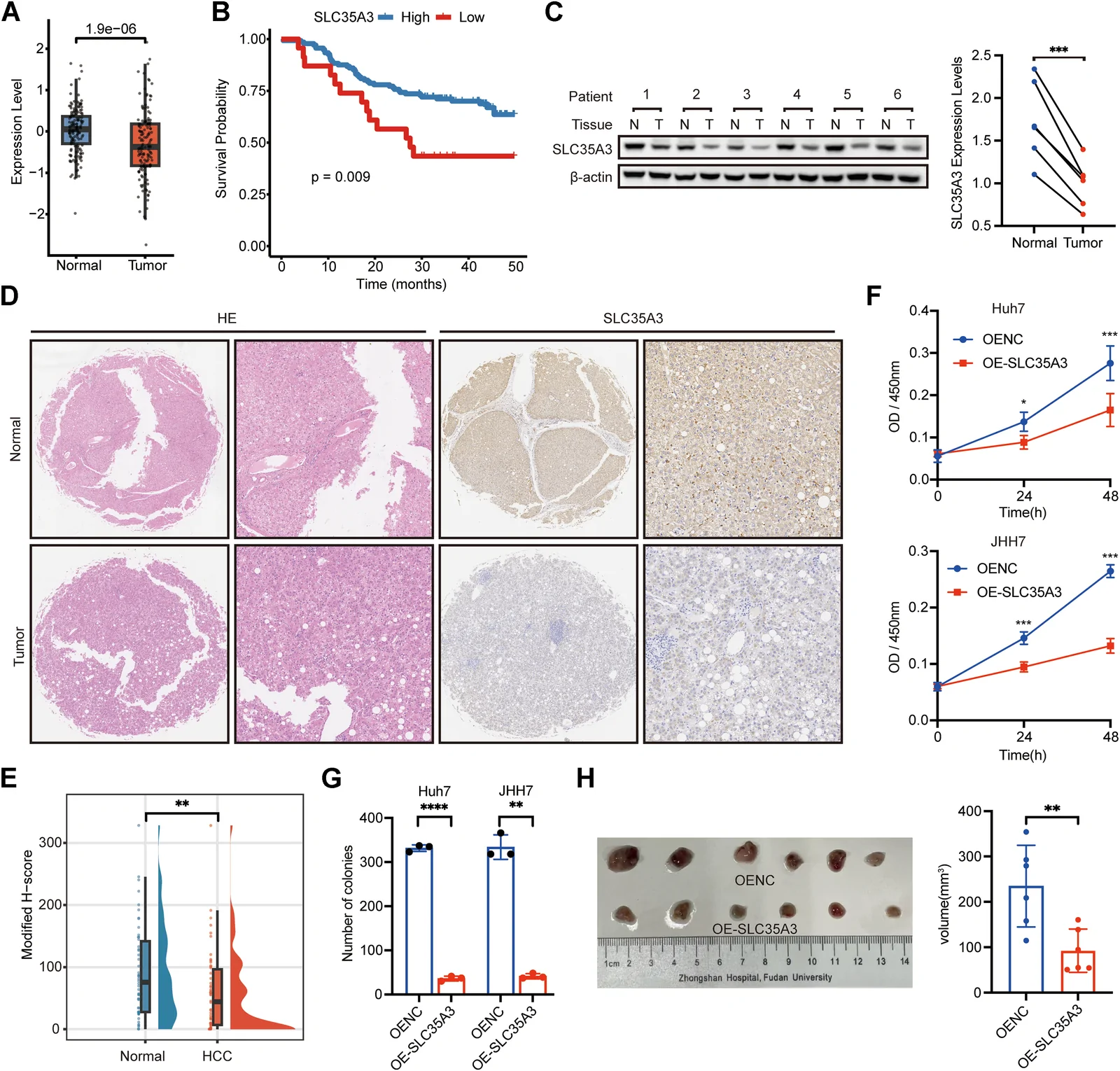
**SLC35A3 is downregulated in HCC and suppresses malignant progression.***A*, Box plot showing SLC35A3 protein abundance in tumor and adjacent normal liver tissues from the CPTAC-HCC cohort. *B*, Kaplan–Meier survival curves stratified by SLC35A3 expression in the CPTAC-HCC cohort. *C*, Western blot analysis (*left*) and densitometric quantification (*right*) of SLC35A3 protein levels in six paired HCC (T, tumor) and adjacent normal (N, normal) liver tissues. Statistical significance was determined by paired Student's *t* test. *D*, representative H&E and SLC35A3 immunohistochemical staining of paired adjacent normal liver tissue (*top*) and HCC tissue (*bottom*) cores from the TMA. Low- and high-magnification views are presented for each staining condition. *E*, Modified H-scores of SLC35A3 staining in 50 paired HCC and adjacent normal liver tissue. Data distribution is shown as raincloud plots with half-violin density, box plots (median, interquartile range), and individual data points. Statistical significance was determined by Wilcoxon signed-rank test. *F*, Cell proliferation of Huh7 and JHH7 cells with SLC35A3 overexpression (OE-SLC35A3) or vector control (OENC), measured by CCK-8 assay at 0, 24, and 48 h. Statistical significance was determined by two-way ANOVA. *G*, Colony formation assay of JHH7 and Huh7 cells with SLC35A3 overexpression (OE-SLC35A3) or vector control (OENC). *H*, Representative images of excised subcutaneous xenograft tumors derived from OENC and OE-SLC35A3 JHH7 cells are shown on the *left*. Tumor volumes at the experimental endpoint are quantified on the *right*. Statistical significance was determined by unpaired Student's *t* test.

To investigate the functional role of SLC35A3 in HCC, we established stable SLC35A3-overexpressing JHH7 and Huh7 cell lines. Western blot analysis confirmed successful SLC35A3 overexpression in both cell lines, with JHH7 cells showing a more pronounced relative increase owing to their lower basal SLC35A3 expression (Supplementary Fig. S2*E*). Cell proliferation and clonogenic capacity were significantly suppressed following SLC35A3 overexpression, as determined by CCK-8 and colony formation assays (Fig. 2, *F* and *G*; Supplementary Fig. S2*F*). Consistently, subcutaneous xenograft assays in nude mice revealed that SLC35A3 overexpression significantly suppressed tumor growth compared with vector controls, without causing obvious changes in mouse body weight (Fig. 2*H*). Together, these findings indicate that SLC35A3 acts as a tumor-suppressive regulator in HCC by inhibiting malignant proliferation and tumorigenicity.

### SLC35A3 Overexpression Increases Golgi-Associated UDP-GlcNAc Levels and Promotes Remodeling of Site-specific N-glycosylation

SLC35A3 has been characterized as a Golgi-resident UDP-GlcNAc transporter that supplies UDP-GlcNAc for Golgi glycosylation reactions. ELISA quantification showed that SLC35A3-overexpressing JHH7 cells contained significantly higher levels of UDP-GlcNAc in Golgi-enriched fractions (Fig. 3*A*). To further characterize the glycosylation changes associated with SLC35A3, we performed site-specific N-glycoproteomic profiling in JHH7 cells with or without SLC35A3 overexpression by LC-MS/MS. In total, we identified 304 glycoproteins and 3820 intact N-glycopeptides, with detailed information provided in Supplementary Table S4.

**Fig. 3:**
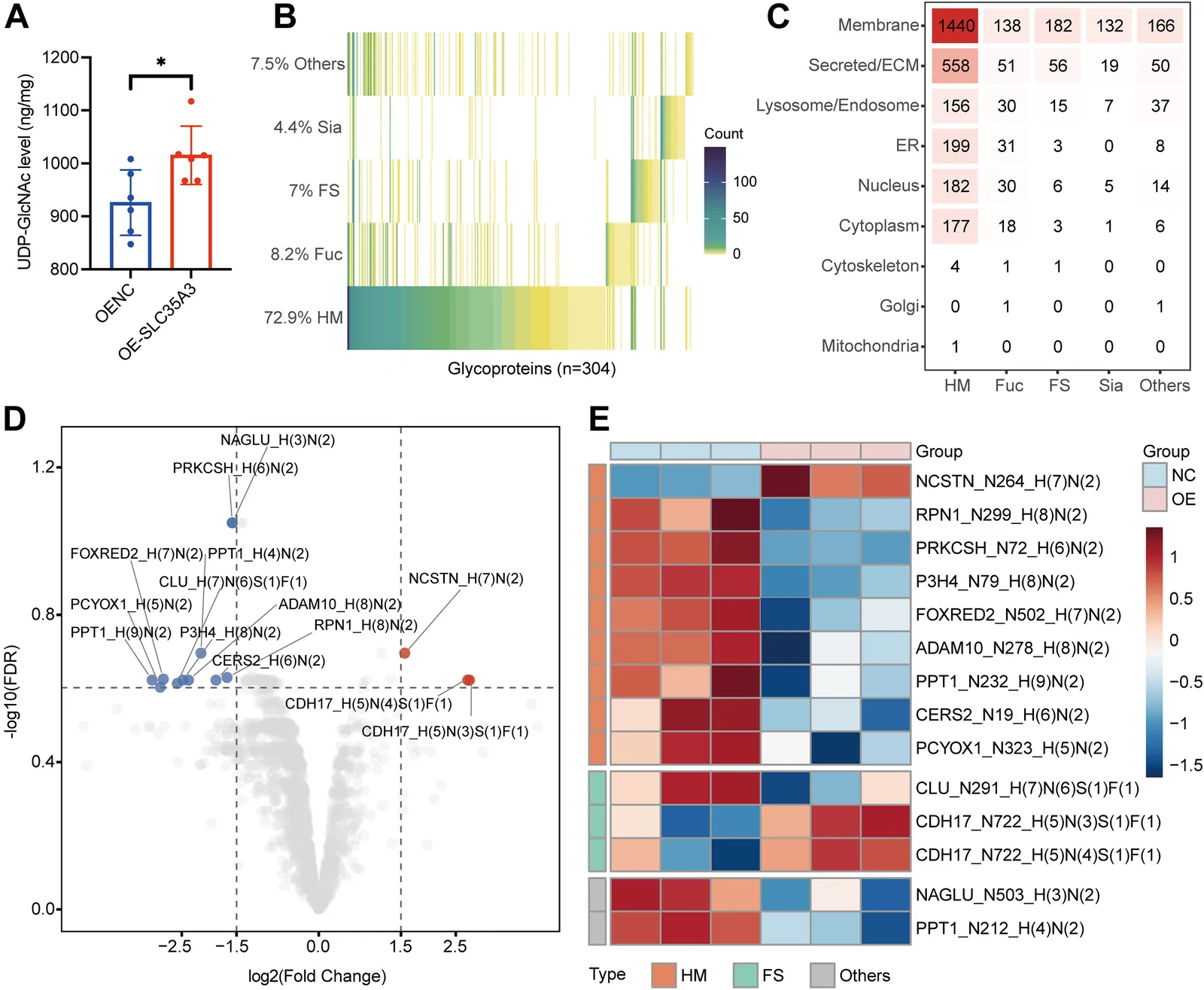
**SLC35A3 restoration enhances Golgi UDP-GlcNAc levels and promotes N-glycan maturation in HCC cells.***A*, ELISA quantification of UDP-GlcNAc in Golgi apparatus of JHH7 cells with SLC35A3 overexpression (OE) and corresponding control cells (NC) (n = 6). Statistical significance was determined by unpaired Student's *t* test. *B*, heatmap showing the number of glycoforms per glycoprotein. Each column represents a glycoprotein, and each row represents a glycoform category. *C*, summary of UniProt database localization categories for each glycoform. Numbers indicate glycopeptide counts for each glycoform. *D*, volcano plot showing differentially expressed glycopeptides between SLC35A3-overexpressing and control JHH7 cells. Significantly altered glycopeptides (fold change >1.5, adjusted *p* < 0.05) are highlighted and labeled with protein name and glycan composition. *E*, heatmap of 14 significantly altered glycopeptides across biological replicates. Rows represent glycopeptides annotated by glycoform, and columns represent samples. Color scale indicates row-normalized glycopeptide abundance.

Glycan compositions comprising N-acetylglucosamine (GlcNAc, N), hexose (H), fucose (F), and sialic acid (S). These N-glycans could be categorized into five types: high-mannose (HM; N = 2, 9 ≥ H ≥ 5, F = 0, S = 0), sialylation-only (Sia; F = 0, S > 0), fucosylation-sialylation (FS; F > 0, S > 0), fucosylation-only (Fuc; F > 0, S = 0), and Others. Among the identified intact N-glycopeptide, HM was the predominant compositional category (72.9%), followed by Fuc (8.2%) (Fig. 3*B*). Analysis of glycosylation distribution revealed substantial N-glycan complexity and microheterogeneity in JHH7 cells, as glycoproteins harbored variable numbers of N-glycosylation sites and individual sites were modified by multiple distinct glycoforms (Supplementary Fig. S3*A*). Motif analysis confirmed that the identified glycosylation-centered peptides were highly enriched for the N-X-S/T sequon, with Thr and Ser occupying the +2 position in 63.73% and 35.38% of sequences, respectively (Supplementary Fig. S3*B*). To further characterize the subcellular distribution of glycoform heterogeneity, identified glycoforms were annotated according to UniProt subcellular localization categories. The majority of glycoforms were derived from membrane and secreted/ECM-associated glycoproteins, consistent with the typical localization of N-glycosylated proteins in the secretory pathway (Fig. 3*C*). In contrast, intracellular compartments including the nucleus, cytoplasm, and mitochondria showed negligible glycosylation. These findings indicate that protein glycosylation is highly enriched in secretory and membrane-associated proteins exposed to the extracellular environment, supporting their established roles in cell–cell communication and microenvironment interactions.

We next performed differential analysis at both the proteomic and glycoproteomic levels. Principal component analysis (PCA) showed that the glycoproteomic dataset enabled a clearer discrimination of the two groups along PC1 compared to proteomic dataset, indicating that N-glycoprotein profiles carry more discriminative information (Supplementary Fig. S3*C*). Protein-level differential analysis identified 14 significantly altered proteins, including SLC35A3, supporting successful SLC35A3 overexpression (Supplementary Fig. S3*D*). Given that glycopeptide abundance can be influenced by the abundance of the corresponding parent proteins, intact N-glycopeptide intensities were further normalized to their matched protein abundances to better characterize glycosylation-specific alterations. Comparative N-glycoproteomics between SLC35A3-overexpressing and control JHH7 cells identified 14 significantly altered glycopeptides (fold change >1.5, *p* < 0.05, FDR < 0.25) (Fig. 3*D*). Although the number of significantly altered glycopeptides was limited, most altered HM-type glycopeptides were decreased after SLC35A3 overexpression, whereas FS-type glycopeptides showed increased abundance (Fig. 3*E*). To obtain a global overview of glycopeptide abundance changes upon SLC35A3 overexpression, we compared the mean normalized glycopeptide intensities between the two groups. Most glycopeptides clustered near the diagonal, indicating an overall conserved glycosylation landscape. Nevertheless, a subset of glycopeptides deviated markedly from the diagonal, indicating selective, site-specific remodeling of N-glycosylation (Supplementary Fig. S3*E*). Thus, SLC35A3 overexpression is associated with selective, site-specific remodeling of the N-glycopeptide landscape, rather than with a nonspecific global shift in glycosylation.

### Molecular Docking Identifies Poliumoside as a Potential SLC35A3 Modulator

Given that SLC35A3 was downregulated in HCC and exhibited tumor-suppressive effects, we next sought to identify small-molecule compounds that could potentially bind to and modulate SLC35A3 activity. As no experimental crystal structure of SLC35A3 has been reported, the AlphaFold-predicted structure (AF-Q9Y2D2-F1) was employed for structure-based virtual screening. Two compound libraries, the MCE Bioactive Compound Library (25,300 compounds) and the MCE 80K Diversity Library (80,000 compounds), were screened using a stepwise Glide docking workflow, as described in detail in the Experimental Procedures. The top-ranked 200 compounds from each library were retained for further evaluation, with detailed docking scores and physicochemical properties provided in Supplementary Table S5.

The top 10 compounds ranked by docking score were labeled as C1-C10, and their chemical structures and docking scores are shown in Supplementary Fig. S4. We used lectin blotting as an initial functional readout to screen compounds that potentially enhance SLC35A3-associated UDP-GlcNAc-dependent glycosylation. Specifically, JHH7 cells were treated with top 10 compounds under identical conditions for 48 h, followed by lectin blotting analysis using ConA, MAL-II, SNA, and AAL. Among the tested compounds, compound 6 (C6, poliumoside, HY-N0033) induced the most prominent increase in multiple lectin-binding signals (Fig. 4, *A–D*). Further validation confirmed that compound 6 significantly increased ConA-, MAL-II-, SNA-, and AAL-binding signals compared with DMSO control (Fig. 4, *E–H*). We then examined the predicted binding mode of poliumoside with SLC35A3. Molecular docking analysis revealed that poliumoside was accommodated within the predicted binding pocket of SLC35A3, forming eight hydrogen-bond interactions with bond distances indicated in the 3D interaction view (Fig. 4, *I–L*).

**Fig. 4:**
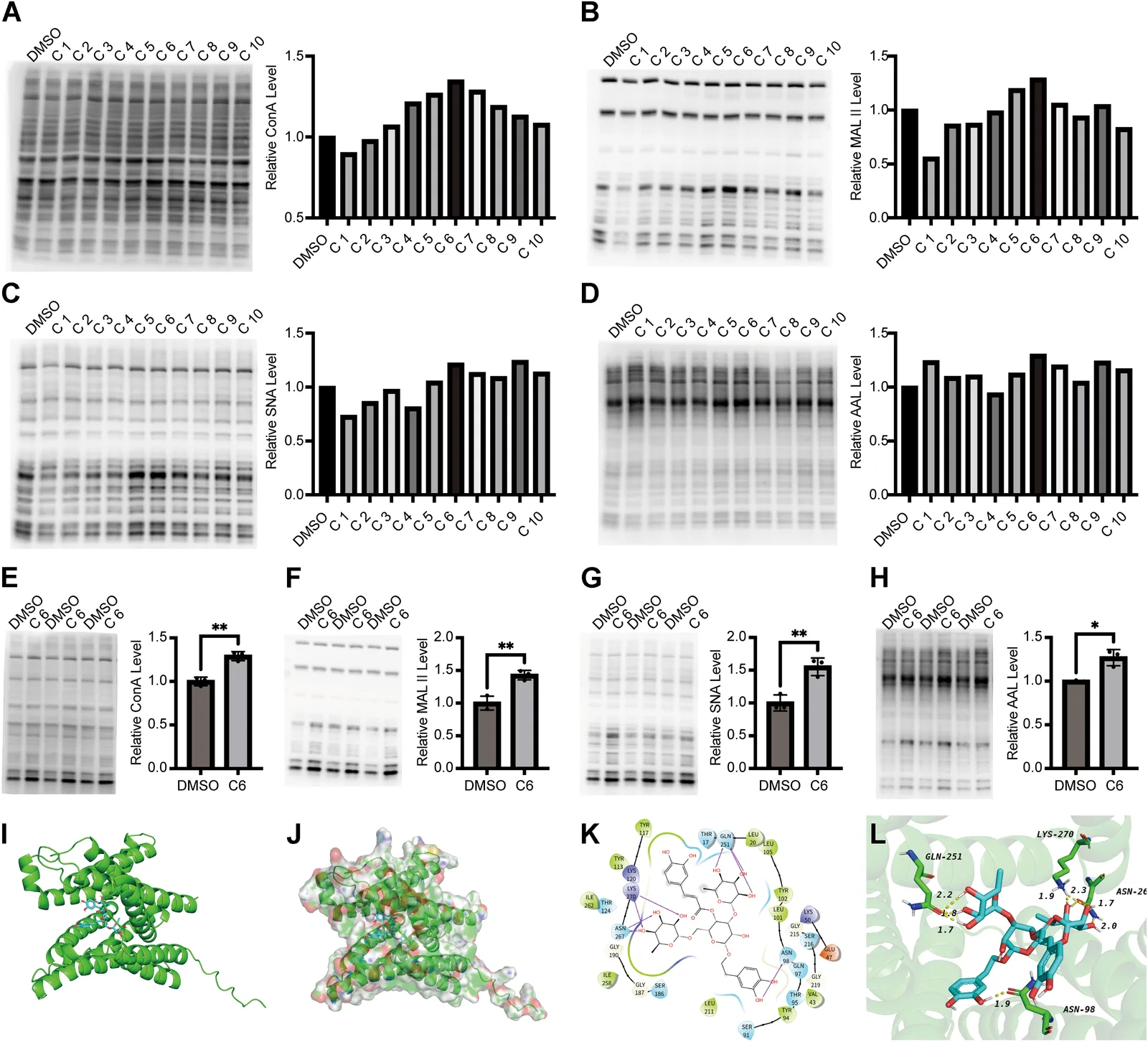
**Molecular docking identifies poliumoside as a potential SLC35A3 modulator.***A–D*, lectin blot analysis of JHH7 treated with DMSO or top 10 candidate compounds (C1-C10). Blots were probed with ConA (*A*), MAL II (*B*), SNA (*C*), and AAL (*D*), followed by densitometric quantification relative to the DMSO control. *E–H*, validation of compound 6 (poliumoside, HY-N0033) in JHH7 cells treated with poliumoside or DMSO vehicle control for 48 h. Lectin blotting was performed using ConA (*E*), MAL-II (*F*), SNA (*G*), and AAL (*H*). The *right panels* show densitometric quantification normalized to the DMSO control. Data are presented as mean ± SD from three independent experiments (n = 3). *I*, cartoon representation of the AlphaFold-predicted SLC35A3 structure (*green*) with poliumoside docked into the predicted binding pocket. *J*, surface representation of SLC35A3 showing the binding pocket with poliumoside. *K*, two-dimensional ligand interaction diagram depicting hydrogen bonds, hydrophobic contacts, and participating residues. *L*, three-dimensional detailed view of poliumoside within the binding pocket, with hydrogen bond distances (Å) indicated by *yellowdashed lines*.

### Poliumoside Suppresses Malignant Phenotypes of HCC Cells

Having identified poliumoside as a candidate modulator of SLC35A3-associated glycosylation phenotypes, we next evaluated whether poliumoside could suppress malignant phenotypes in HCC models. CCK-8 assays showed that poliumoside reduced the viability of JHH7 cells in a dose-dependent manner, with an IC_50_ of 3.648 μg/ml (95% CI: 2.852–4.661 μg/ml; Fig. 5*A*). Consistently, colony formation assays demonstrated that poliumoside markedly impaired the clonogenic growth of HCC cells (Fig. 5*B*). In addition, flow cytometric analysis revealed that poliumoside treatment increased the proportion of apoptotic cells, suggesting that poliumoside-induced growth inhibition may be accompanied by enhanced apoptosis (Fig. 5*C*). We further investigated the anti-tumor effect of poliumoside *in vivo*. Compared with the PBS control group, poliumoside treatment significantly slowed xenograft tumor growth, as evidenced by reduced tumor volume over time (Fig. 5*D*). At the endpoint, tumors from the poliumoside-treated group were visibly smaller and exhibited significantly lower tumor weights than those from the control group. Together, these results demonstrate that poliumoside suppresses HCC progression both *in vitro* and *in vivo*, providing proof-of-concept for pharmacological modulation of SLC35A3-mediated glycosylation in HCC.

**Fig. 5:**
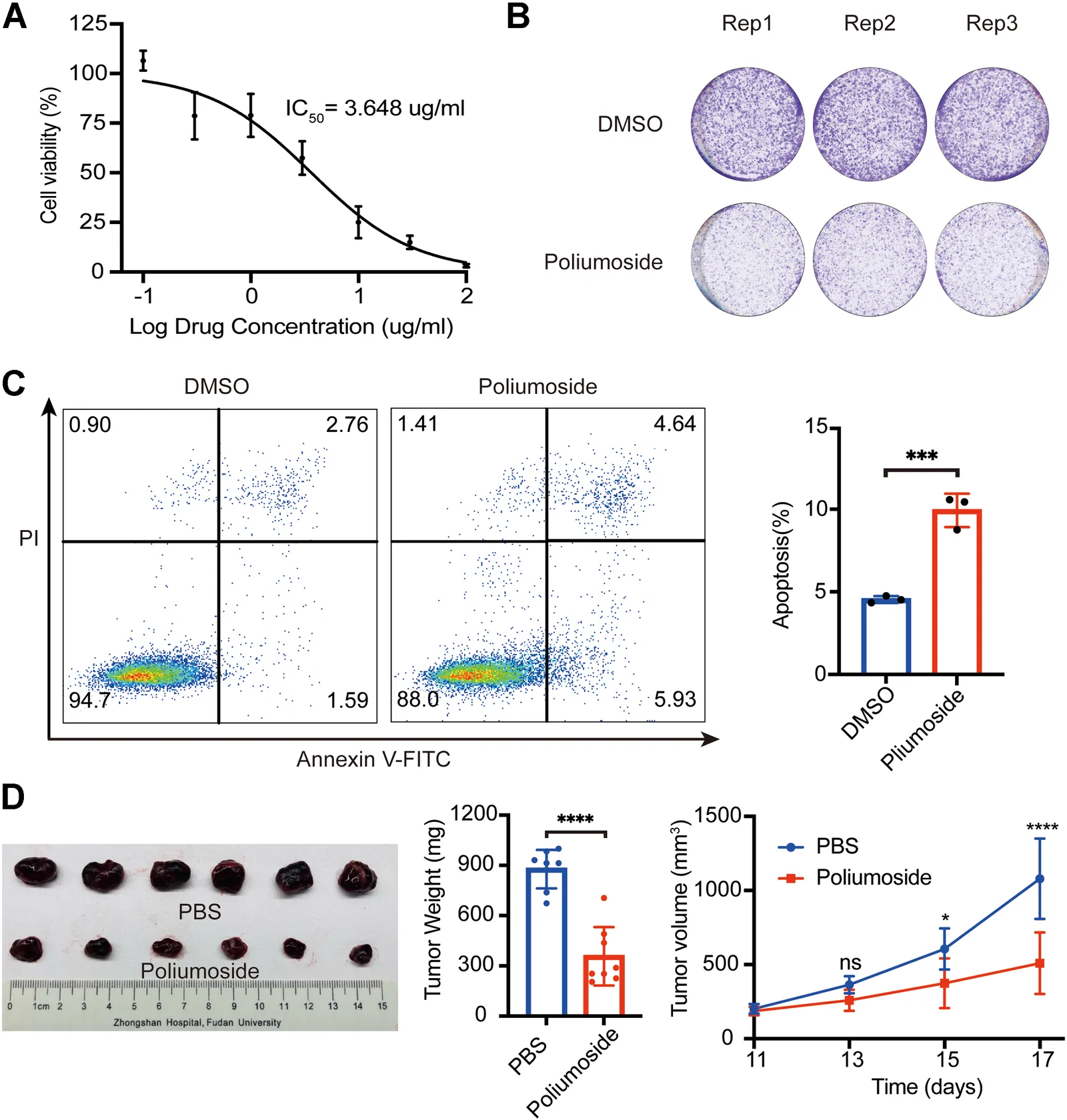
**Poliumoside suppresses malignant phenotypes of HCC cells.***A*, Dose–response curve of poliumoside in JHH7 cells. Cell viability was assessed by CCK-8 assay. Data were fitted to a nonlinear regression model to calculate IC50. *B*, colony formation assay of JHH7 cells treated with poliumoside. *C*, flow cytometric analysis of apoptosis in JHH7 cells treated with DMSO or poliumoside (10 μM, 48 h). *Left panels* show representative Annexin V-FITC/PI staining plots; *right panel* shows quantification of apoptotic cells (Annexin V+). *D*, *in vivo* antitumor efficacy of poliumoside in subcutaneous xenograft model. *Left*, representative images of excised tumors at the study endpoint; *middle*, tumor weights at the study endpoint; *right*, tumor volumes measured over time.

## Discussion

HCC remains one of the most lethal malignancies worldwide, with limited therapeutic options for advanced-stage patients. Aberrant tumor-associated glycosylation represents an emerging class of therapeutic targets that remain underexploited in cancer drug discovery. In this study, we used an integrative proteomic, prognostic, structural ligandability, and glycosylation-related screening strategy to prioritize SLC35A3 as a candidate tumor suppressor in HCC. Our main findings are fourfold. First, SLC35A3 expression was lower in HCC than in paired normal liver tissues in large-scale proteomic cohorts and validation samples, and lower expression was associated with unfavorable prognosis. Second, restoring SLC35A3 expression reduced the proliferative and clonogenic capacity of JHH7 and Huh7 cells and limited subcutaneous xenograft growth in nude mice. Third, SLC35A3 overexpression increased UDP-GlcNAc abundance in Golgi-enriched fractions and was accompanied by site-specific N-glycopeptide changes characterized by a relative reduction in HM glycoforms and an increase in FS glycoforms. Fourth, poliumoside was identified by structure-based virtual screening and lectin-based functional screening as a compound associated with increased glycosylation-related signals and inhibition of HCC cell and xenograft growth. Taken together, these data support SLC35A3-associated Golgi glycosylation as a candidate tumor-suppressive axis in HCC.

The mechanistic basis for SLC35A3-mediated tumor suppression lies in its essential role as a UDP-GlcNAc transporter. UDP-GlcNAc is synthesized in the cytosol, and its transport into the Golgi lumen is required to supply donor substrates for GlcNAc-dependent glycosylation reactions. In the Golgi apparatus, UDP-GlcNAc serves as an obligate donor substrate for N-acetylglucosaminyltransferases, including enzymes involved in the formation of branched complex-type N-glycans. The essential role of UDP-GlcNAc transport in N-glycan maturation is evolutionarily conserved. In plants, loss of the Golgi UDP-GlcNAc transporter UGNT1 abolishes complex-type N-glycans, yielding exclusively high-mannose structures (23). It is noteworthy that SLC35A3 is not the sole UDP-GlcNAc transporter in mammalian cells. Other transport systems, including SLC35D2 and the recently characterized TMEM241, have also been implicated in Golgi UDP-GlcNAc transport 24, 25. Nevertheless, SLC35A3 appears to function as an effective and rate-limiting supplier for specific glycosylation reactions, particularly complex N-glycan maturation. Consistent with this concept, Slc35a3-knockout mice exhibit reduced tri- and tetra-antennary branching with concomitant high-mannose accumulation in brain tissues (26). In line with the requirement for Golgi UDP-GlcNAc transport in complex N-glycan processing, our glycoproteomic profiling of SLC35A3-restored cells revealed a broad reduction in HM glycoforms and selective increases in specific FS glycoforms.

Our findings support a tumor-suppressive role for SLC35A3 in HCC. Notably, reduced SLC35A3 expression and its tumor-suppressive function have also been reported in colorectal cancer, suggesting that SLC35A3 may have broader tumor-suppressive relevance in gastrointestinal cancers (27). We hypothesized that small molecules capable of binding SLC35A3 might stabilize a transport-competent conformation, enhance transporter-associated function, or promote SLC35A3-dependent Golgi glycosylation without necessarily increasing SLC35A3 expression. Based on this rationale, we performed structure-based virtual screening and identified poliumoside as a candidate compound predicted to bind SLC35A3. Functionally, poliumoside enhanced glycosylation-related phenotypes, suggesting potential modulation of SLC35A3-mediated glycosylation.

Nevertheless, several limitations should be acknowledged. Although molecular docking suggested a potential interaction between poliumoside and SLC35A3, direct target engagement has not yet been experimentally validated. In addition, we did not directly assess SLC35A3 transporter activity or UDP-GlcNAc transport across the Golgi membrane due to technical limitations. Despite these limitations, our findings provide initial evidence that poliumoside may influence the SLC35A3-associated glycosylation axis and suggest that targeting SLC35A3 may represent a potential upstream strategy for modulating aberrant glycosylation in HCC. Further mechanistic studies are warranted to determine whether the effects of Poliumoside are directly mediated by SLC35A3. Overall, our findings nominate SLC35A3-associated Golgi glycosylation as a potentially actionable vulnerability in HCC and provide a rationale for further exploration of pharmacological strategies targeting this pathway.

## Data Availability

The raw mass spectrometry data have been deposited in GlycoPOST under accession number GPST000756 (http://doi.org/10.50821/GLYCOPOST-GPST000756). The processed data supporting the conclusions of this study are provided in Supplementary Tables S1-S5. The analysis code is available from the corresponding author upon reasonable request.

## Supplemental data

This article contains supplemental data.

## Conflict of interest

The authors declare that they have no conflicts of interest with the contents of this article.
