# Supplementary material for: SLC35A3 Remodels Site-Specific N-Glycosylation and Defines a Druggable Glycosylation Target in Hepatocellular Carcinoma
Source: Mol Cell Proteomics. 2026 Oct 5;25(11):101672. doi: 10.1016/j.mcpro.2026.101672 (PMC13743935; doi:10.1016/j.mcpro.2026.101672)
Supplement: Supplementary file 1 [file mmc6.docx]

**Supplementary Materials**

**Supplementary Fig. S1. Total ion chromatograms (TICs) of JHH7 samples analyzed by LC-MS/MS for N-glycopeptide profiling.**

(A) TICs of three negative-control (NC) samples.

(B) TICs of three SLC35A3-overexpressing (OE) samples.

**Supplementary Figure S2. Additional characterization of SLC35A3 in HCC.**

(A) Box plot showing SLC35A3 protein abundance in tumor and adjacent normal liver tissues from the Zhongshan-HCC cohort.

(B) Kaplan-Meier survival curves stratified by SLC35A3 expression in the Zhongshan-HCC cohort.

(C) Radar plot comparing SLC35A3 immunohistochemical staining features between HCC tissues and paired normal liver tissues in the TMA.

(D) Distribution of SLC35A3 staining intensities in HCC tissues and paired normal liver tissues in the TMA. Staining intensity was classified as negative, weak, moderate, or strong.

(E) Western blot showing SLC35A3 protein levels in JHH7 and Huh7 cells stably expressing SLC35A3 (OE-SLC35A3) or vector control (OENC). β-Actin served as the loading control.

(F) Images of colony formation assays in OENC and OE-SLC35A3 JHH7 and Huh7 cells.

**Supplementary Figure S3. N-glycoproteomic landscape following SLC35A3 overexpression in JHH7 cells.**

(A) Stacked bar plots show the distribution of N-glycosylation sites per glycoprotein (left panel), the distribution of identified N-glycans per glycoprotein (middle panel), and the distribution of distinct N-glycans per N-glycosylation site (right panel).

(B) Motif analysis of identified N-glycosylation sites (left) and distribution of amino acids at the +2 position relative to the glycosylated asparagine residue (right).

(C) Principal component analysis (PCA) of proteomic (upper panel) and N-glycoproteomic (lower panel) profiles from OENC and OE-SLC35A3 JHH7 cells.

(D) Volcano plot of differentially expressed proteins between OE-SLC35A3 and OENC JHH7 cells.

(E) Scatter plot comparing mean normalized glycopeptide intensities between OENC and OE-SLC35A3 JHH7 cells. Each dot represents an intact N-glycopeptide, colored by glycoform.

**Supplementary Fig. S4. Information of the top 10 compounds ranked by docking score.**

**Supplemental Table S1. Prioritization of candidate target proteins for HCC.**

**Sheet 1: Prognosis-associated protein candidates from CPTAC-HCC cohort.** List of candidate proteins significantly down‑regulated in HCC relative to adjacent non‑tumor liver tissues from the CPTAC‑HCC cohort, with prognostic metrics derived from univariate Cox proportional‑hazards regression analysis. Column definitions: Gene_Symbol, HGNC gene symbol; log2FC, log2-transformed fold change in protein abundance between HCC tumor tissues and the adjacent normal liver tissues; DE_FDR, false discovery rate (FDR)-adjusted significance value from differential-expression analysis; HR, hazard ratio from univariate Cox regression; HR_95CI, 95% confidence interval of hazard ratio; Cox_pvalue, p-value of univariate Cox regression.

**Sheet 2: Prognosis-associated protein candidates from Zhongshan-HCC cohort.** List of candidate proteins significantly down‑regulated in HCC relative to adjacent non‑tumor liver tissues from the Zhongshan-HCC cohort, with prognostic metrics derived from univariate Cox proportional‑hazards regression analysis. Columns are defined identically to Sheet 1.

**Sheet 3: Prioritized HCC-associated protein targets ranked by Relevance Score.** Column definitions: Gene_Symbol, HGNC gene symbol; Description, gene name from GeneCards; Uniprot_ID, UniProt accession number; Relevance_Score, GeneCards-derived disease-gene relevance score; higher values indicate stronger reported association with HCC.

**Supplemental Table S2. P2Rank-based ligand-binding pocket prediction for HCC candidate targets.**

Column definitions: UniProt_ID, UniProtKB protein accession number; Gene_Symbol, HGNC gene symbol; Pocket_ID, unique pocket identifier for each protein; Pocket_Rank, pocket rank sorted by P2Rank score; P2Rank_Score, raw machine-learning pocket-ligandability score; Calibrated_Probability, calibrated probability (0-1) that the pocket is a real ligand-binding site; High_Confidence_Ligandable_Candidate, Yes/No; Yes if protein has ≥1 pocket with calibrated probability > 0.9; SAS_Points, number of solvent-accessible-surface points forming the pocket; Surface_Atom_Count, number of surface atoms assigned to the pocket; Pocket_Center_X/Y/Z, pocket-center Cartesian coordinates (Å) from AlphaFold model; Pocket_Residues, lining residues of the pocket.

**Supplemental Table S3.** **QuPath-derived quantitative immunohistochemical scoring of SLC35A3 in paired HCC and adjacent normal liver tissue microarray cores.**

Column definitions: Patient_ID, anonymized patient identifier; TMA_Core, identifier for an individual tissue spot (core) on the tissue microarray; Total_Cells, total number of cells detected and included in the TMA Core. Negative_Cells, Weak_Cells, Moderate_Cells, and Strong_Cells denote the numbers of cells classified as negative, weak, moderate, or strong for SLC35A3 staining intensity, respectively. Positive_Cells_Percent denotes the percentage of positively stained cells among all detected cells. Modified_H_Score denotes the modified histochemical score calculated as: 1 × percentage of weakly stained cells + 2 × percentage of moderately stained cells + 3 × percentage of strongly stained cells. Allred_Proportion denotes the Allred proportion score based on the percentage of positive cells: 0, no positive cells; 1, <1%; 2, 1-10%; 3, 11-33%; 4, 34-66%; and 5, ≥67%. Allred_Intensity denotes the predominant staining-intensity score: 0, none; 1, weak; 2, moderate; and 3, strong. Allred_Score denotes the sum of Allred_Proportion and Allred_Intensity, with a possible range of 0–8. Higher modified H-scores and Allred scores indicate stronger SLC35A3 IHC positivity.

**Supplemental Table S4. Site-specific intact N-glycopeptide quantification in negative-control and SLC35A3-overexpressing JHH7 cells.**

Column definitions: Glycopeptide_ID, unique identifier for each intact N-glycopeptide; Peptide, identified peptide backbone sequence; ProSites, assigned N-glycosylation site position; GlycanComposition, monosaccharide composition; GlyID, glycan identifier; Intensity_NC_1/2/3, MS signal intensities in the three negative-control replicates; Intensity_OE_1/2/3, MS signal intensities in the three SLC35A3-overexpressing replicates. Scan denotes the tandem mass spectrometry (MS/MS) scan number used for identification. RT denotes chromatographic retention time. PrecursorMH denotes the precursor mass reported as the protonated molecular mass [M+H]^+^, PrecursorMZ denotes the precursor mass-to-charge ratio, and Charge denotes the precursor-ion charge state. TotalScore denotes the overall glycopeptide identification score, whereas PepScore and GlyScore denote the peptide-assignment and glycan-assignment scores, respectively. TotalFDR, PeptideFDR, and GlycanFDR denote the estimated FDRs for the overall glycopeptide assignment, peptide component, and glycan component, respectively.

**Supplemental Table S5. Virtual screening and molecular docking results for candidate SLC35A3-binding compounds.**

Column definitions: Rank, compound ranking in the docking; Catalog_Number, supplier catalog identifier; CAS_Number, Chemical Abstracts Service (CAS) registry number; Molecular_Formula, elemental composition of the compound; Molecular_Weight, molecular mass of the compound; Docking_Score, predicted binding score; Ligand_Efficiency, docking score normalized by ligand size; LogP, the predicted or calculated octanol/water partition coefficient; H_Bond_Acceptors, number of hydrogen-bond acceptor atoms; H_Bond_Donors, number of hydrogen-bond donor atoms; Rotatable_Bonds, number of rotatable single bonds; SMILES, Simplified Molecular Input Line Entry System representation of the compound structure.
